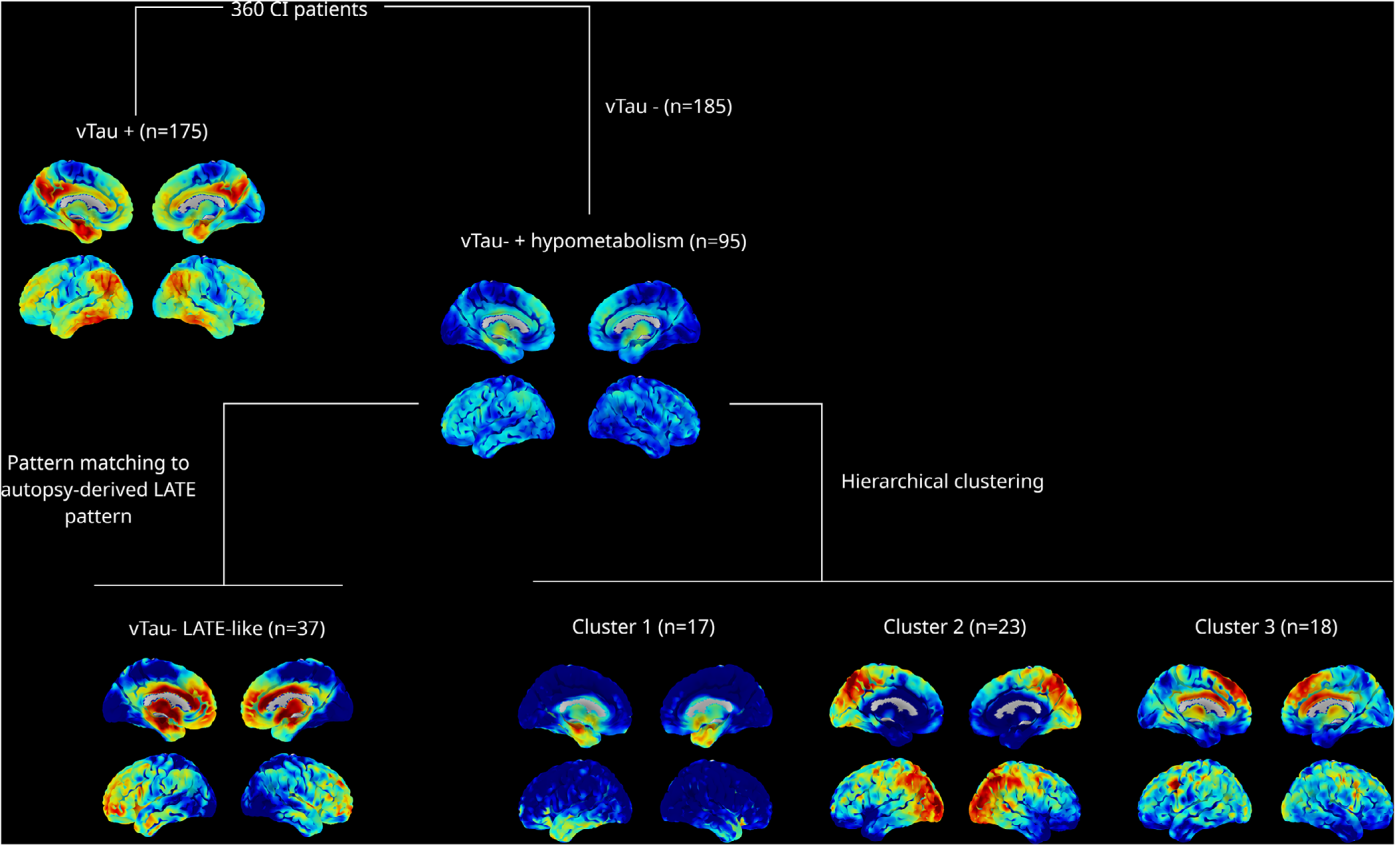# FDG‐PET patterns of LATE & co: disentangling the pathological heterogeneity of Tau‐negative amnestic syndromes

**DOI:** 10.1002/alz.088748

**Published:** 2025-01-09

**Authors:** Jesús Silva‐Rodríguez, Alexis Moscoso, Miguel Labrador‐Espinosa, Pascual Sanchez‐Juan, Michael Schöll, Michel J. Grothe

**Affiliations:** ^1^ Reina Sofia Alzheimer Center, CIEN Foundation, ISCIII, Madrid, Madrid Spain; ^2^ Wallenberg Centre for Molecular and Translational Medicine, University of Gothenburg, Gothenburg Sweden; ^3^ Reina Sofia Alzheimer Centre, CIEN Foundation, ISCIII, Madrid Spain

## Abstract

**Background:**

About 20‐30% of clinically diagnosed AD dementia patients do not meet pathologic criteria for AD and this proportion is even higher in amnestic MCI. Among tau‐negative amnestic patients, limbic‐predominant age‐related TDP‐43 encephalopathy (LATE) has been described as a principal diagnostic alternative, especially at advanced age. LATE is characterized by a specific temporo‐limbic hypometabolic signature on FDG‐PET that may aid in differential diagnosis. However, little is known about the prevalence of this and other hypometabolism patterns among tau‐negative amnestic patients.

**Method:**

360 cognitively impaired subjects (261 aMCI, 99 ADD) from the ADNI study who underwent ^18^F‐Flortaucipir Tau‐PET and ^18^F‐FDG‐PET were divided into Tau+ and Tau‐ using a visual read method. After screening Tau‐ patients for regional hypometabolism, LATE‐like hypometabolism patterns were identified using an automated pattern‐matching approach based on pathology‐specific FDG‐PET signatures derived from autopsy‐confirmed cases. FDG‐PET patterns among Tau‐ patients not fitting the LATE‐specific pattern were further examined using a hierarchical clustering approach. Group differences in age, hippocampal volume, and cognitive variables were studied.

**Result:**

185 patients (162 aMCI, 23 ADD) were visually rated as Tau‐. Among those presenting any regional hypometabolism (N=95), 37 patients (39%) had a LATE‐like hypometabolism pattern (Figure 1). Hierarchical clustering of the remaining individuals revealed 3 different clusters suggestive of other neurodegenerative conditions, including a left‐lateralized temporal pole pattern reminiscent of semantic dementia (C1, 18%), a posterior‐occipital pattern characteristic for DLB (C2, 24%), and a more diffuse frontal pattern (C3, 19%). Compared to the other Tau‐ groups, the LATE‐like group was significantly older (p=0.02) and had more pronounced memory impairment (p=0.02) and hippocampal atrophy (p=0.03) (Figure 2). When initially screening Tau‐ patients for older age (>75y) and hippocampal atrophy, the majority of patients showed a LATE‐like FDG‐PET pattern (59%), but other patterns remained to be present in this population at smaller proportions (19%, 9%, and 13%, for C1, C2, and C3, respectively).

**Conclusion:**

FDG‐PET provides valuable diagnostic information after a negative Tau‐PET scan. Among older tau‐negative patients with hippocampal atrophy, temporo‐limbic hypometabolism suggestive of LATE is the most prevalent pattern, but distinct hypometabolic patterns suggestive of other neurodegenerative conditions are also present in non‐negligible proportions.